# Formation, structure and dynamics of hydroxyl nest defects in zeolites

**DOI:** 10.1039/d6cp00804f

**Published:** 2026-08-04

**Authors:** Alec W. Desmoutier, Jingcheng Guan, Jamal Abdul Nasir, Thomas W. Keal, You Lu, C. Richard A. Catlow, Alexey A. Sokol

**Affiliations:** a University College London, Kathleen Lonsdale Materials Chemistry 20 Gordon Street London WC1H OAJ UK uccades@ucl.ac.uk; b School of Chemistry, Cardiff University, Park Place Cardiff CF10 3AT UK; c Scientific Computing Department, STFC, Daresbury Laboratory Daresbury Warrington WA4 4AD UK

## Abstract

Hydroxyl nest defects in zeolite frameworks, formed through the removal of T-sites followed by hydrolysis, are of critical importance for catalytic applications due to their high reactivity and role in stabilizing metal species. This study employs hybrid QM/MM calculations to characterize single hydroxyl nests in several zeolite frameworks, focusing on formation energies and structures and on the atomic-level mechanisms governing proton dynamics. In particular, we distinguish between proton rotation around oxygen atoms and proton transfer between different hydroxyl sites. Using Nudged Elastic Band (NEB) calculations, we determine the energy barriers for both mechanisms in chabazite. Our results reveal a negative (favourable) formation energy for these defects in ZSM-5, faujasite and quartz (in chabazite it is close to zero) and show that the framework geometry significantly affects the energetics of proton mobility, with rotation often favoured in constrained environments. Structural data and bond angle analysis are reported for each framework. These findings contribute to the understanding of proton behavior in defective zeolites and offer insights for tailoring catalytic properties through defect engineering.

## Abstract 2

QM/MM simulations show hydroxyl nest formation is exothermic in ZSM-5, FAU and quartz (CHA near-neutral), while subsequent proton rotation is favoured over inter-oxygen transfer, with a barrier nearly 40% lower (0.77 *vs.* 1.29 eV).

## Graphical abstract

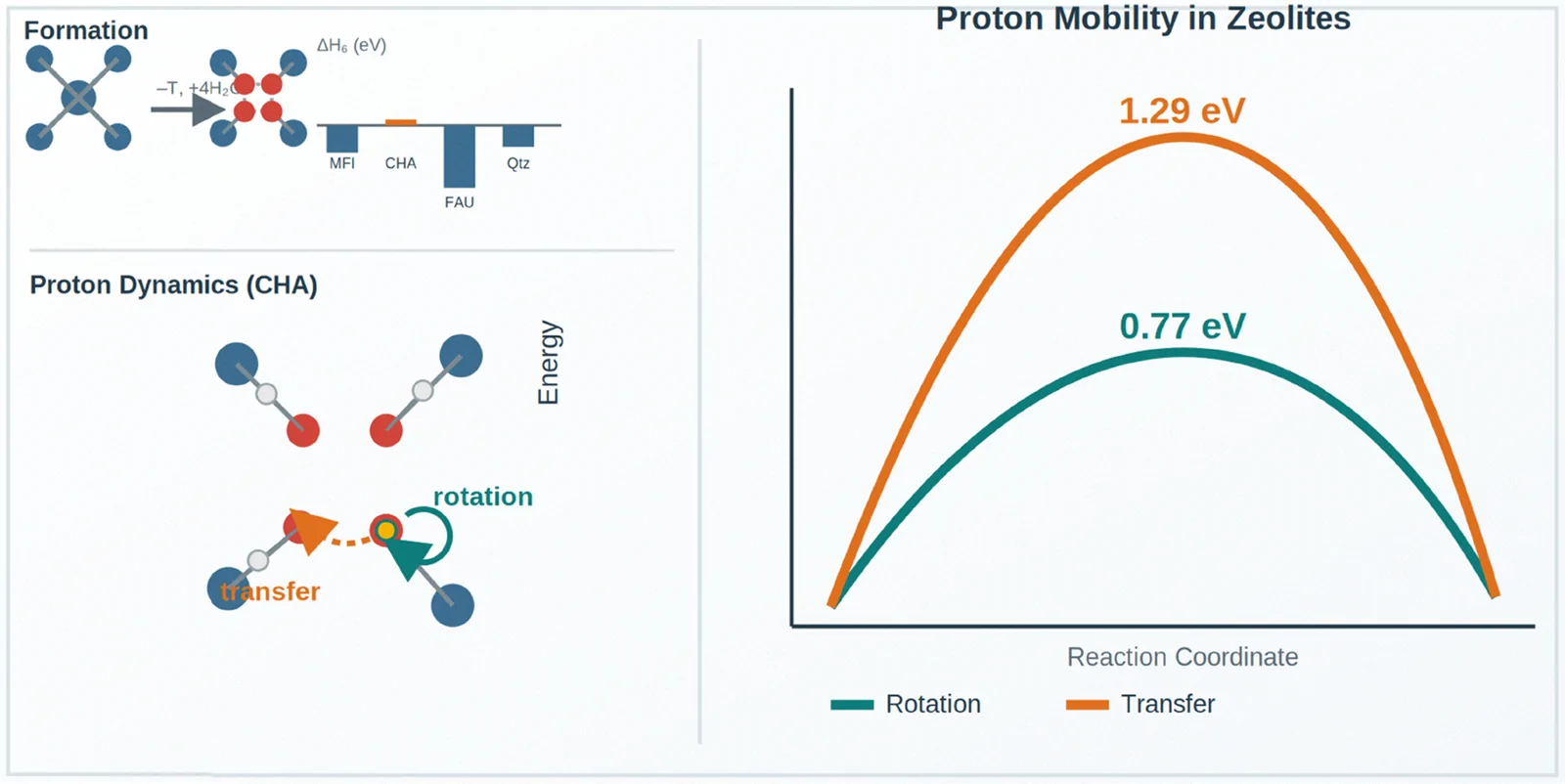

## 1. Introduction

Zeolites are crystalline microporous aluminosilicates that play a central role in modern industrial catalysis, gas separation, and ion exchange technologies.^1^ Their success stems from their unique structural features: a three-dimensional framework of corner-sharing TO_4_ tetrahedra (T = Si or Al), forming well-defined channels and cages on the molecular scale. These features enable shape-selective catalysis and high surface area adsorption, making zeolites indispensable in petroleum refining, petrochemical synthesis, and increasingly in biomass conversion and environmental catalysis.^2–4^ A cornerstone of their catalytic activity lies in the presence of Brønsted acid sites, which arise when trivalent Al^3+^ substitutes for Si^4+^ in the framework, creating a negative charge that is locally compensated by a proton.

**Fig. 1:**
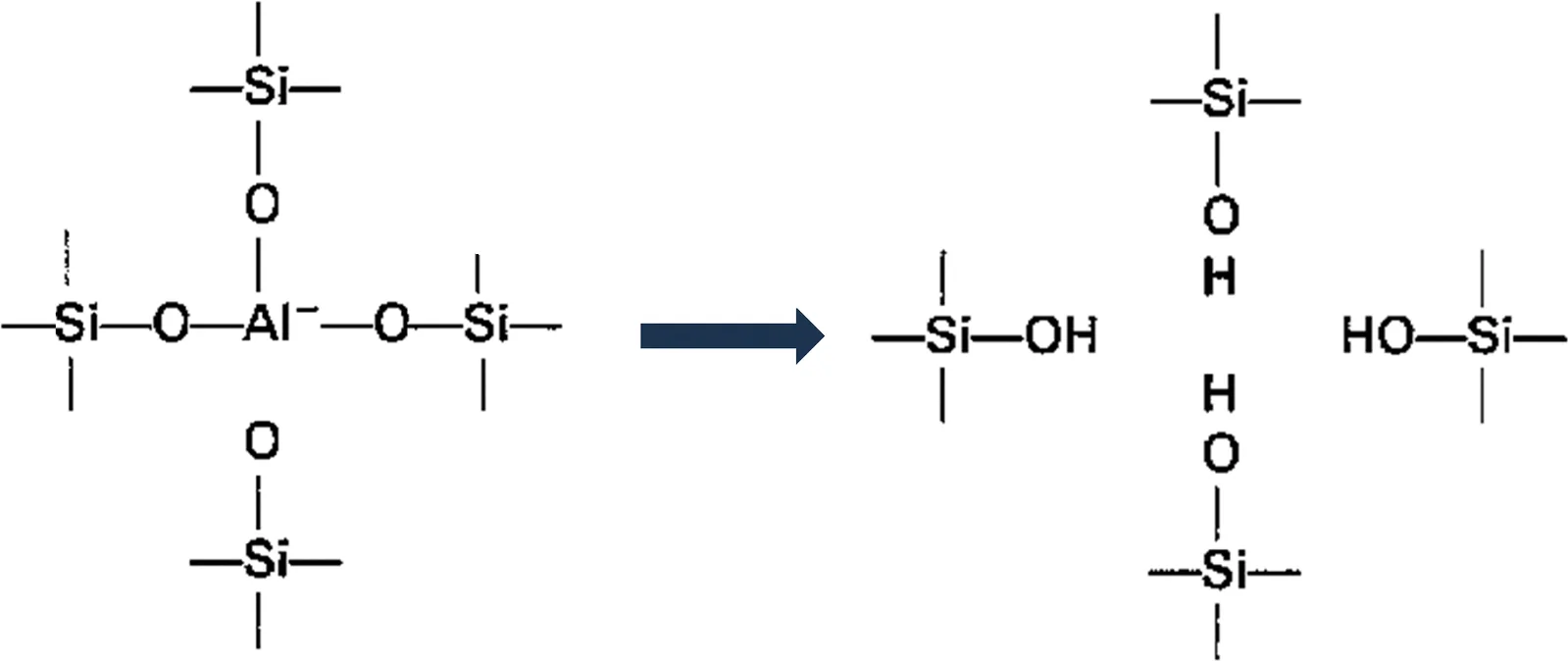
Dealumination process for the formation of hydroxyl nest defects. Shows a depiction of the defectless tetrahedral framework removing Al to form a hydroxyl nest defect.

While the traditional view of zeolite acidity focuses on bridging hydroxyl groups (≡Si–OH–Al≡), real zeolite materials – particularly those subjected to steaming, dealumination, or other forms of chemical treatment – often contain a range of structural defects.^5,6^ One important and increasingly recognized class of such defects is the hydroxyl nest. These are local disruptions in the framework composed of a T site vacancy with protonation of the four surrounding oxygens, as shown schematically in Fig. 1. Hydroxyl nests can form during synthesis, through post-synthetic modifications, or under catalytic conditions, and their presence has been confirmed experimentally by IR spectroscopy, solid-state NMR, and neutron scattering.^7–9^ Some work^10,11^ has suggested that internal silanols of hydroxyl nests may be stable up to 400 °C in defect nests of completely dealuminated zeolites.

Hydroxyl nests are not merely passive byproducts of framework degradation; they can fundamentally alter the physicochemical properties of zeolites.^12–14^ Clusters of silanol groups can engage in hydrogen bonding networks, enhance water adsorption, modulate local polarity, and influence proton mobility.^15^ In some cases, they may even generate new types of acid sites or facilitate cooperative interactions with nearby Brønsted or Lewis acidic centres.^16^ Understanding the structure and dynamics of these defects is therefore critical for the rational design of defect-engineered zeolites with tailored catalytic or adsorption properties.

The exceptional acid catalytic properties of zeolites have been largely attributed to the presence of Brønsted acid centres. The microporous structure of zeolites not only facilitates catalytic activity but also imparts shape selectivity, thereby controlling the nature of the reactions and the resulting products. This unique characteristic of shape-selective acid catalysis has driven intensive research into the properties of Brønsted acid centres within these materials. Both theoretical and experimental techniques have been employed to gain deeper insights. For instance, Schroder *et al.*^17^ utilized Mott–Littleton techniques to characterize the Al–OH centre in Zeolite Y, achieving vibrational frequencies of the OH group that align well with experimental data. Additionally, quantum mechanical methods have been extensively applied to model the behaviour of acid centres in zeolites and their interactions with small molecules, as demonstrated by the influential work of Sauer^18^ and Gale *et al.*^19^ These studies collectively enhance our understanding of the fundamental mechanisms underlying zeolite catalysis and guide the development of more effective catalytic materials.

Among the various types of silanol defects, single hydroxyl nests in which four silanol groups surround a vacant framework T-site represent the simplest vacancy defect. They can arise from isolated hydrolysis events or be present in high-silica zeolites that lack extensive defect clustering. Single hydroxyl nests are of particular interest because they represent a minimal perturbation to the framework while still introducing new chemical functionality. Moreover, they serve as good models for studying proton behaviour in confined, polarizable environments.

A key aspect of silanol behaviour in zeolites, particularly in the context of catalytic or thermal conditions, is the mobility of the associated protons. Two primary modes of motion can occur: proton rotation, in which the O–H vector reorients around the fixed silanol oxygen, and proton transfer, in which the proton hops between neighboring oxygen atoms in the framework, potentially forming transient hydrogen bonds or shared-proton configurations. While these processes may appear similar from a spectroscopic standpoint, they differ markedly in their energetic profiles, spatial extent, and chemical consequences. Proton transfer can lead to transient formation of new Brønsted acid sites or influence the local acid strength, while both types of motion contribute to configurational entropy and may modulate dipolar interactions or vibrational signatures. Disentangling these two types of motion is thus necessary for correctly interpreting experimental data and for understanding how silanol defects influence the dynamic properties of zeolites.

In this study, we investigate the fundamental behaviour of single hydroxyl nest defects in representative zeolite frameworks, with particular emphasis on identifying and characterising the energetic and structural features and how these influence the relative energies of proton rotation and proton transfer. By probing these fundamental defect processes, we aim to shed light on the broader implications of hydroxyl nests in zeolite chemistry, particularly in relation to acidity, reactivity, and framework dynamics in realistic, defect-rich environments.

## 2. Methodology

All hybrid quantum mechanics/molecular mechanics (QM/MM) calculations were carried out using the ChemShell^20,21^ computational framework, which interfaces NWChem^22^ for the QM region and DL_POLY^23^ or GULP^24^ for MM interactions. The goal was to model isolated single hydroxyl nest defects in zeolite frameworks and study the energetics and mechanisms of proton rotation and transfer within these confined environments. We modelled proton nests in ground state silica material α quartz and three siliceous zeolites: faujasite (FAU), ZSM-5 (MFI) and chabazite (CHA). All were modelled with purely siliceous compositions. The model employed in this study consists of a quantum mechanical (QM) region of 146 (CHA), 111 (α quartz) 139 (MFI), 107 (FAU) atoms surrounding the vacancy site, embedded within a significantly larger 25 Å environment treated using classical molecular mechanics (MM). This QM/MM approach provides an accurate representation of the local active site while overcoming the computational cost associated with large supercell calculations required in periodic density functional theory (DFT) calculations to mitigate artefacts arising from periodic image interactions. Additionally, the non-periodic nature of the QM/MM framework facilitates the use of hybrid DFT functionals to enhance the accuracy of the electronic structure description, as implemented in the present work. The surrounding electrostatic environment is efficiently captured through a combination of MM atoms and point charges, as described in detail below.

### 2.1. Cluster construction and defect generation

**Fig. 2:**
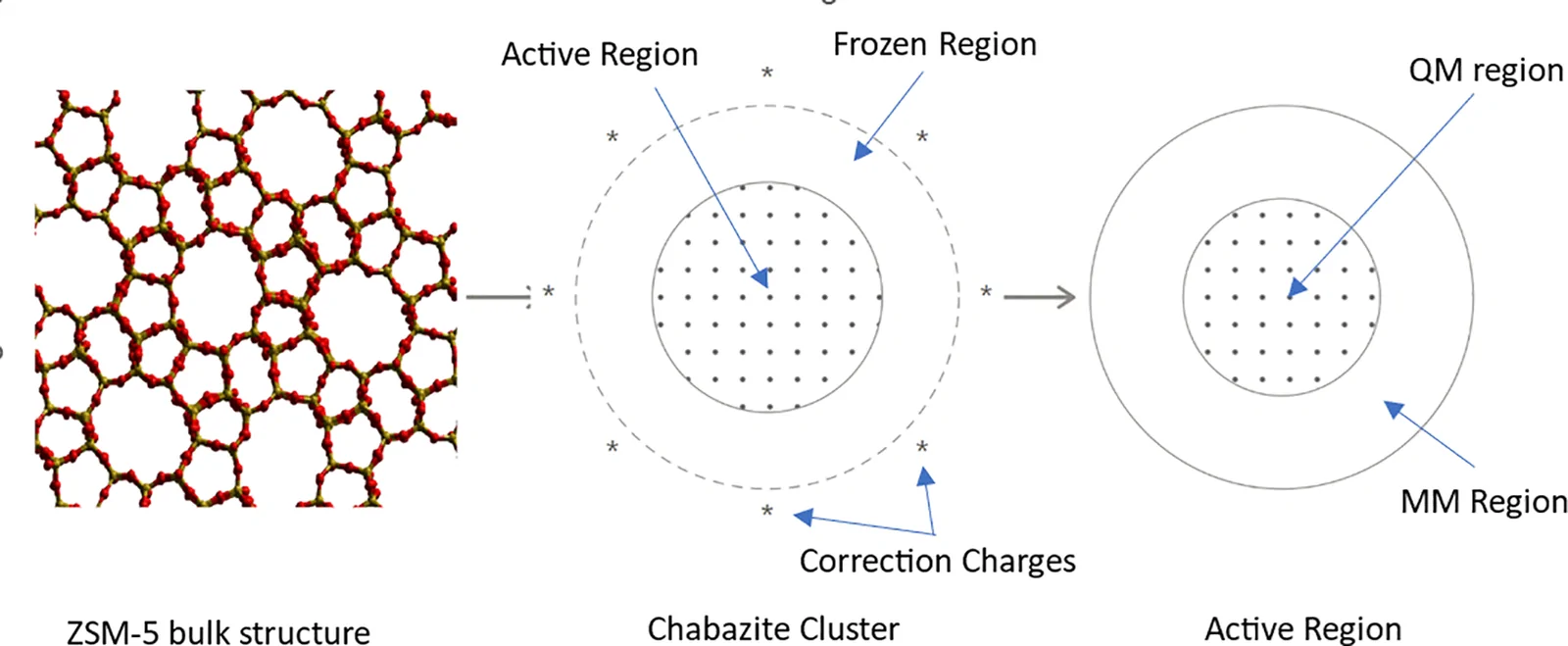
Generation of finite cluster model for ChemShell applications.^25^

The hydroxyl nest was introduced into representative alpha quartz and siliceous zeolite frameworks of ZSM-5 (MFI), FAU, and CHA types by removing a single T-site (typically Si) and subsequently protonating the four adjacent oxygen atoms to form four terminal Si – OH groups. The unit cells structure was acquired from IZA database where the atom coordinates and the cell parameters were optimized with Distance Least-Squares (DLS76) geometric refinement assuming a pure SiO_2_ composition. These unit cells were subsequently duplicated to form larger clusters from which a sphere of diameter 60 Å was cut out. At the extremity of the clusters, point charges were introduced to represent the electrostatic interaction with the bulk material. Cluster models were extracted from the fully periodic frameworks and centered on the defect site. Dangling bonds at the QM/MM boundary were capped using link atoms (in this case hydrogen). This entire process is depicted in Fig. 2. The active molecular mechanics region encompassed atoms within a ∼25 Å radius around the QM zone and included ∼1500–2000 atoms, treated with point charges. The active region is in turn surrounded by a frozen spherical MM layer. The T site selected for this investigation was the site found at the intersection between both major rings found in CHA, FAU and ZSM-5, however the position of α-quartz was simply picked at the centre of the cluster due to its uniformity.

### 2.2. QM/MM embedding and force field parameters

The MM region was modeled using the Hill–Sauer force field,^26^ which is well validated for silicate systems. Charges for MM atoms were assigned based on the scaled original parametrization: +1.2 for Si and −0.6 for O.^27^ In a QM/MM setup, electrostatic interactions between the QM and MM regions are typically handled using an electrostatic embedding scheme, which allows the QM electron density to respond to the surrounding MM environment. The MM embedding included both mechanical and electrostatic coupling to the QM region. The boundary between regions was carefully optimized to avoid artificial polarization, and convergence with respect to cluster size and MM buffer region was verified in benchmark tests. Boundary energies arise where a QM region is cut away from the bulk by breaking covalent bonds. In zeolite this process is carried out where the QM region is terminated by oxygen atoms, in order to reduce the artificial interactions between regions. When the QM region is cut off the terminating oxygen atoms are capped with link atoms (hydrogen atoms) then calculations are performed with these link atoms to simulate a larger region. A charge correction is applied to the MM region to offset the loss of oxygen atoms.

### 2.3. Quantum mechanical calculations

All QM calculations were performed using DFT with the B3LYP exchange–correlation functional.^28^ Geometry optimizations employed the def2-TZVP basis set and a smaller def2-SVP basis set on outer QM silicon and oxygen atoms. The higher def2-TZVP^29^ was used for the central QM region (hydroxyl nest site and the coresponding adjacent T-sites). A convergence criterion of 1 × 10^−5^ Ha was applied to the total energy and 0.0045 Ha/Bohr to the atomic forces.

### 2.4. Proton motion pathways and NEB calculations

Proton transfer and rotation mechanisms were modeled using the nudged elastic band (NEB)^30^ method implemented in DL-FIND.^31^ Each path was constructed using 10 intermediate images, linearly interpolated between fully optimized initial and final states. These images were optimized simultaneously using the climbing-image NEB algorithm until the force on each image was less than 0.0045 Ha/Bohr. The resulting minimum energy paths (MEPs) were used to extract energy barriers for proton transfer and rotation.

For rotational mechanisms, the proton remained covalently bonded to the same oxygen atom, while for transfer pathways, a bond-breaking and bond-forming event occurred between neighboring silanols. In both cases, NEB paths were analyzed for symmetry, saddle point location, and reaction coordinate metrics (O–H distances, O⋯H distance, and Si–O–H angles).

## 3. Results and discussion

We first consider the formation energies and structures of the hydroxyl nest defects, noting that previous studies using a wide range of methods have calculated the formation energies of hydroxyl nests at −2.4 to 1.8 eV.

### 3.1. Formation and structural characteristics of single hydroxyl nest defects in zeolites

The optimized geometries and energetics of the single hydroxyl nest defects in the four siliceous frameworks: ZSM-5 (MFI), chabazite (CHA), faujasite (FAU), and α-quartz reveal key differences in defect energies and morphology, driven by the topological and steric constraints of each host framework.

### 3.2. ZSM-5 (MFI)

**Fig. 3:**
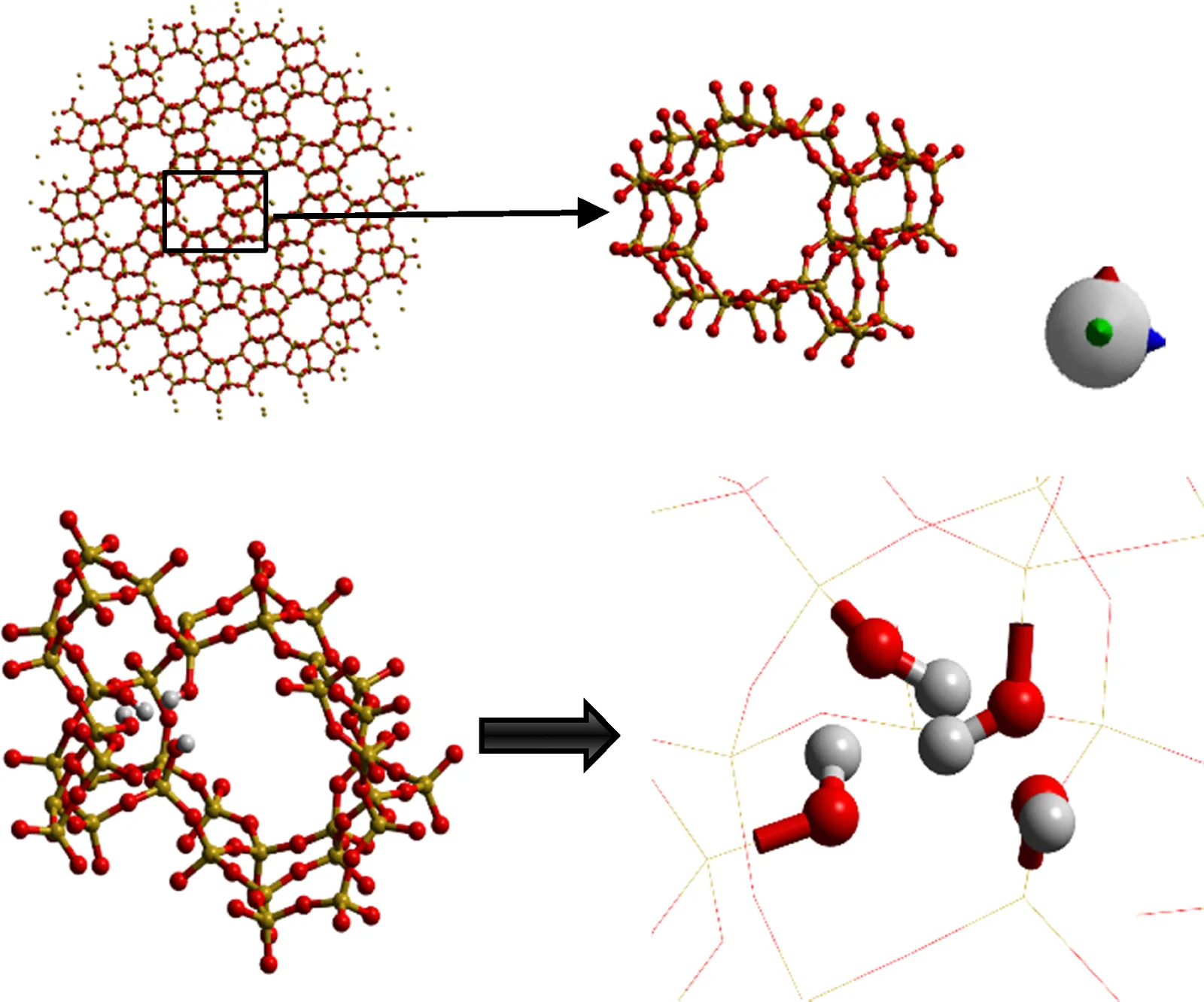
The QM/MM setup: the full pure ZSM-5 cluster without 3D perspective (top left) with the quantum mechanical region (top right). The outermost region contains point charges to ensure the Madelung potential in the central region of the cluster to accurately reproduce bulk. Modified QM/MM cluster after the embedding setup process showing the entire QM region utilised of ZSM-5 (bottom left) followed by a close up of the Hydroxyl nest defect site (bottom right). Atom colour codes: Si: yellow, O: red and H: white.

ZSM-5 exhibits an interconnected system of 5- and 10-membered rings, with moderate spatial constraints around the T-sites. Upon removal of a single silicon atom and subsequent protonation, the four resultant silanol groups adopt a near-tetrahedral configuration, as shown in Fig. 3. Optimization gave an average O–H bond length of 0.97 Å and hydrogen bonding distances ranging from 1.80 to 2.80 Å, indicative of moderate hydrogen bonding among the silanols. The Si–O–H angles averaged 109.5°.

**Fig. 4:**
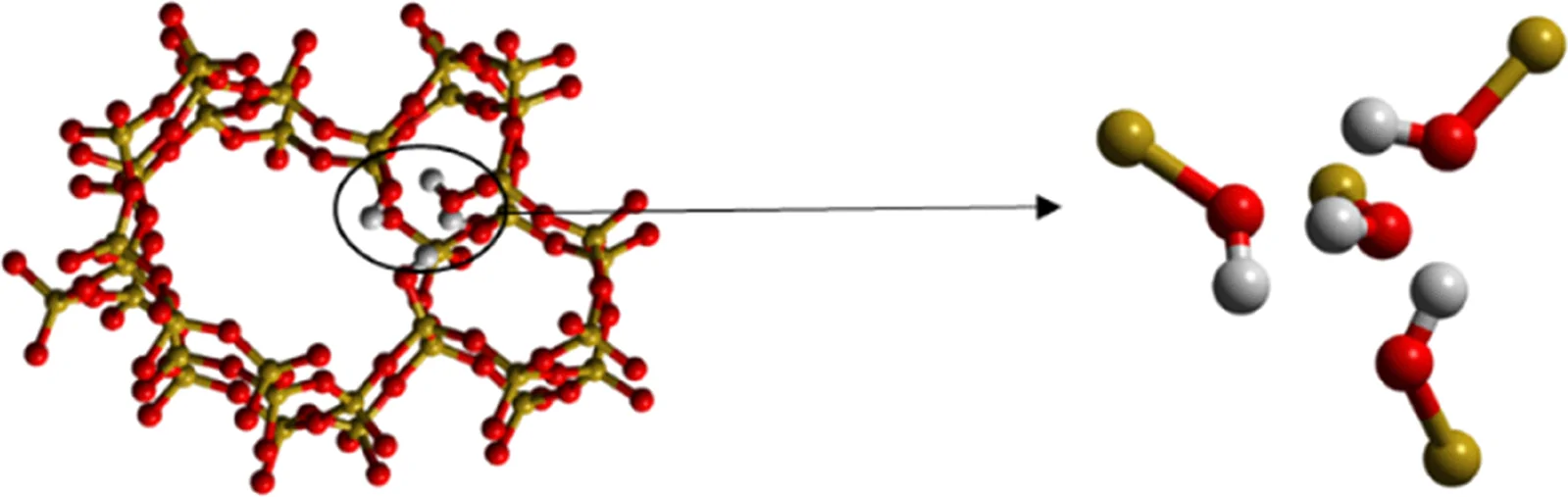
The defect geometry of ZSM-5, showing the characteristic 3-membered ring silanol cluster with intra-cluster H-bonding on the left.

The formation enthalpy of the hydroxyl nest in ZSM-5 was computed to be –0.25 eV relative to the fully siliceous framework and gas-phase water molecules using the equation below.*E*_f_ = *E*(defect) + *E*(by product) − *E*(pure) − *E*(reactants)*E*(defect) is the energy of the cluster with the introduced defect (ZSM-5 cluster with hydroxyl nest defect site included), *E*(byproduct) is the energy of the atoms removed from the zeolite cluster (the removal of silicon to form the byproduct which we take as orthosilicic acid – Si(OH)_4_), *E*(pure) the energy of a pure silicious ZSM-5 cluster and *E*(reactants) is the energy of the four water molecules used to protonate the defect site once silicon with four hydroxyl groups is removed. The energies of these Si(OH)_4_ and H_2_O molecules in a vacuum using NWChem under ChemShell and def2-TZVP basis set are recorded as −593.12 Ha for Si(OH)_4_ and −76.45 Ha for H_2_O. This is the first QM/MM calculation of the hydroxyl nest defect formation enthalpy in ZSM-5, which has previously only been studied using semi-classical, semi-empirical and *ab initio* periodic techniques (Fig. 4).

### 3.3. Chabazite (CHA)

**Fig. 5:**
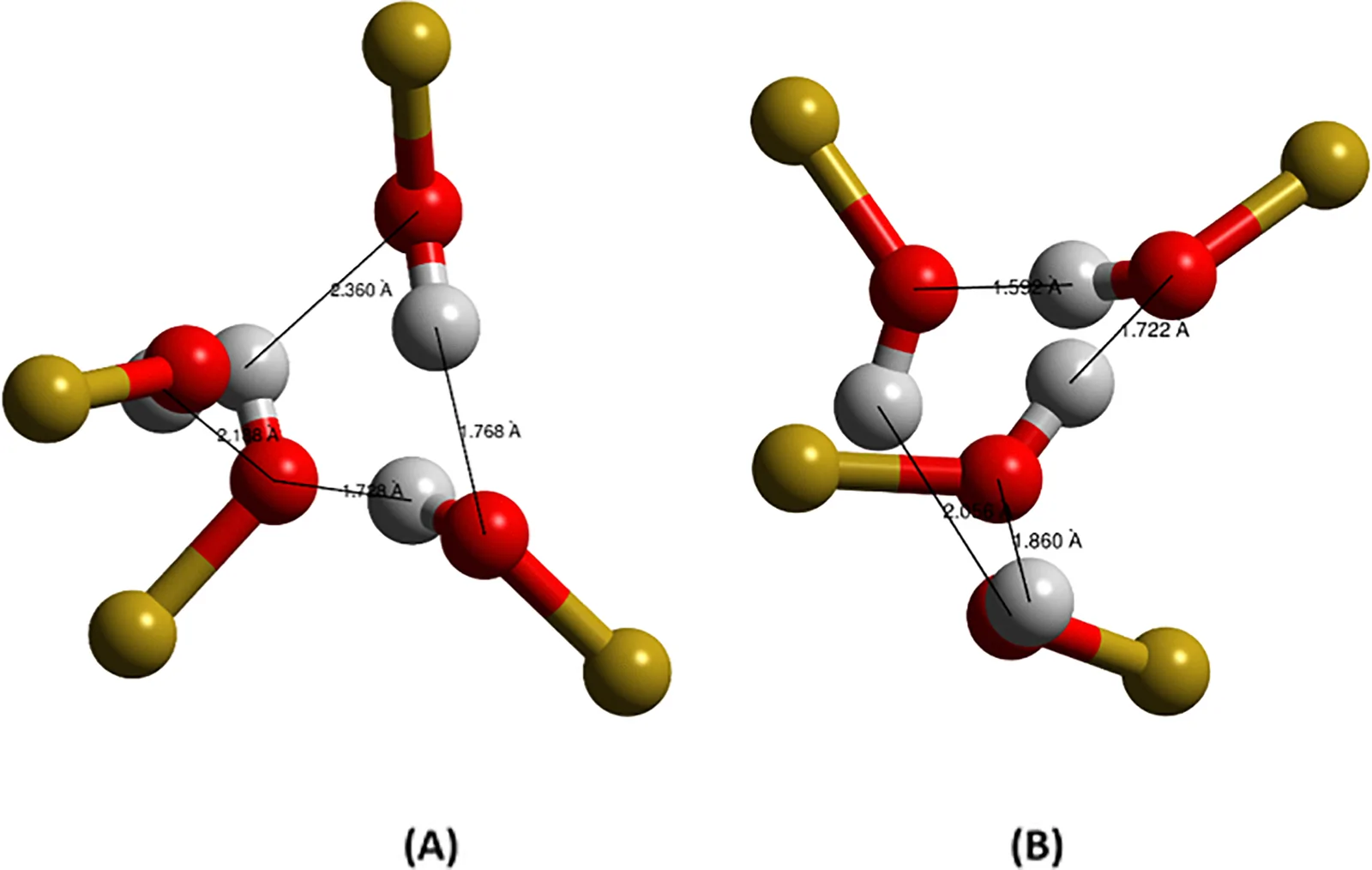
The 4-membered silanol arrangement in CHA (A) and FAU (B) viewed along the crystallographic axis, highlighting the compact defect core and the symmetry of the hydroxyl nest.

Chabazite, a small-pore zeolite with 6- and 8-membered rings, presents a more confined environment for defect formation. Removal of a silicon T-site within the double six-membered ring (D6R) unit leads to the formation of a planar triangular array of silanol groups with a singular silanol adjacent to the plannar array, as shown in Fig. 5(A), similar to the MFI case as described above. The geometry optimization showed shorter hydrogen bond distances (1.8–2.6 Å) and smaller O–Si–O angles, reflecting the framework's greater rigidity and the enforced proximity of the silanol groups.

The O–H bond lengths remained ∼0.97 Å, and the constrained triangular configuration facilitated strong hydrogen bonding among the silanols. The calculated formation enthalpy was notably less exothermic than in ZSM-5, at 0.03 eV, which is consistent with the more rigid framework imposed by the CHA topology.

### 3.4. Faujasite (FAU)

The FAU framework, characterized by large 12-membered ring super cages, allows greater spatial separation between the defect-forming oxygens. Upon silicon removal, the silanols adopt an irregular tetrahedral geometry with hydrogen bonding, as shown in Fig. 5(B). The average hydrogen bonding was comparatively smaller (1.6–2.1 Å), and the hydrogen bonding network was correspondingly expected to be stronger. It does not establish the three-membered hydrogen-bonded silanol ring, but a 4-membered ring as is formed.

Owing to the high local flexibility, this open configuration resulted in a more favourable formation enthalpy of –0.61 eV.

### 3.5. α-Quartz

α-Quartz, a dense and rigid siliceous phase, was also examined for comparison. Unlike the zeolitic frameworks, α-quartz exhibits little internal void space, and defect formation induces significant local strain. The silanol groups formed upon Si removal are closely packed together within the framework forcing hydrogen bonding to occur.

The results from the closely packed T-sites are reflected in the computed formation enthalpy of −0.20 eV, the least favourable among the systems studied apart from CHA. The constraints are also reflected in the hydrogen bond distances (1.6–2.2 Å). Unlike the previously discussed clusters, α-quartz lacks the extensive channel system, resulting in greater framework strain exerted on the defect. This strain forces the silanol groups in the cluster to remain in close proximity, giving the cluster a more cuboid appearance due to the constrained arrangement of hydrogen bonds, which promotes the formation of hydrogen bonding between oxygen and the two closest hydrogen atoms.

### 3.6. Comparative analysis

Previous theoretical studies on hydroxyl nest defect formation have reported a wide range of energies, reflecting strong dependence on the computational approach and model choice. Semiclassical methods, based on interatomic potentials tended to overestimate formation energies, with the lowest reported value being 1.02 eV,^32^ while periodic local density approximation (LDA) studies sometimes over bind the defect^33^ obtaining negative formation energies of ∼−2.4 eV relative to interstitial water and ∼−0.5 eV relative to free water. Previously reported values therefore span both positive and negative ranges and highlight the inconsistency between cluster and periodic models, as well as the functionals employed. Early calculations, limited by computational resources, relied on PW91-DFT with periodic boundary conditions and DSOLID/Dmol^3^ implementations of LDA and GGA, leading to significant discrepancies over whether the defect formation process is exothermic or endothermic. In this context, our own results for sodalite utilising a test cluster with QM region consisting of 20 atoms, considering defect formation as the addition of four isolated water molecules to the framework so that orthosilicic acid can be formed, yield a value of −0.37 eV (−35.4 kJ mol^−1^), which lies within the previously reported range but still deviates by over 2 eV compared to some studies.

The optimized defect structures in ZSM-5 and α-quartz clearly illustrate the role of framework topology in directing silanol packing. ZSM-5, FAU, CHA, and quartz support the formation of triangonal rings, hydrogen-bonded nests, to produce more compacted, uncoordinated defects.

The formation of hydroxyl nests is exothermic, except for CHA, where it is very slightly endothermic. The significant variation in energies can be rationalized by considering both the geometric constraints (which favours defect proximity and H-bonding) and the framework's ability to accommodate local distortions. The triangular nest in FAU and quartz represents an optimal geometry for stabilization, while CHA incurs strain energy penalty.

## 4. Proton dynamics in single hydroxyl nest defects: rotation *vs.* transfer

**Fig. 6:**
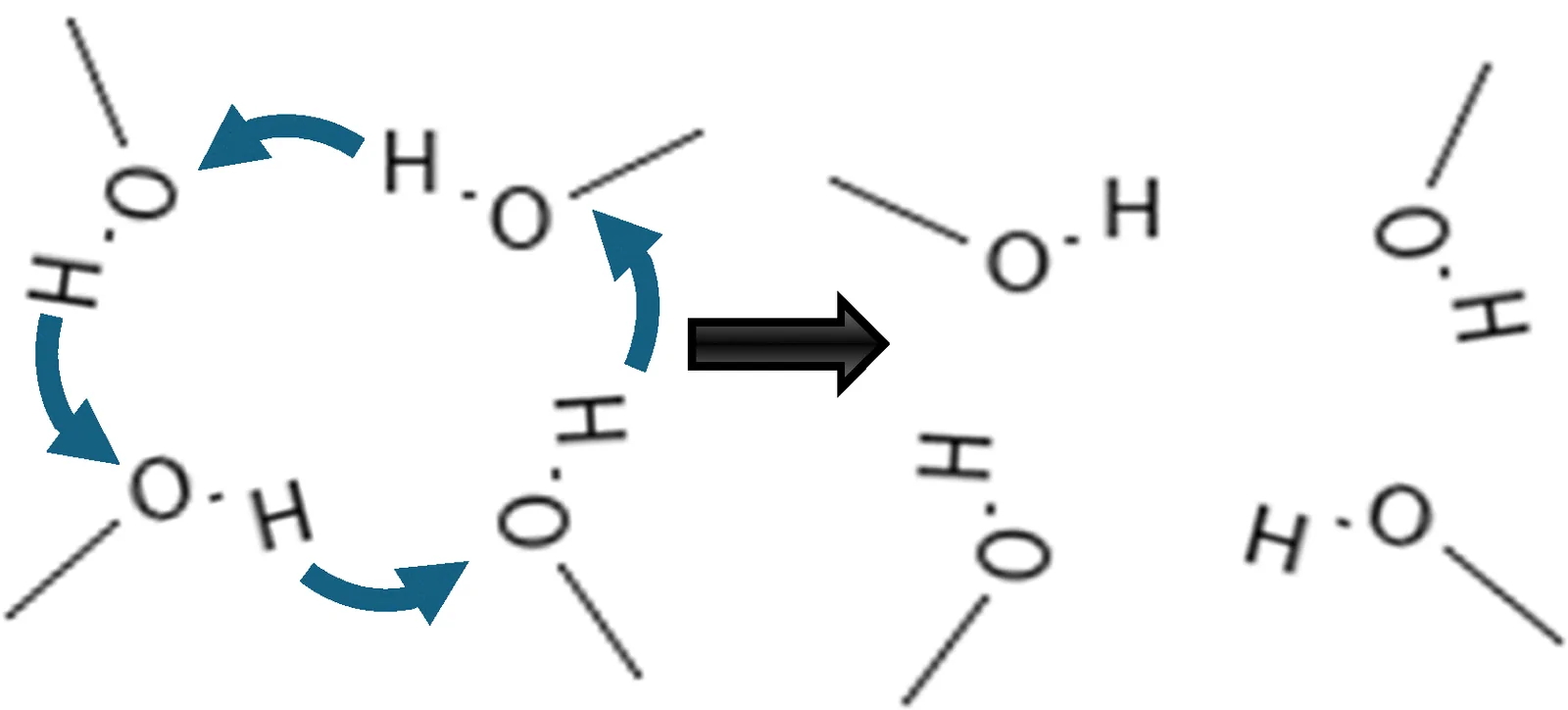
Illustration of the motion of protons within hydroxyl nest defect in zeolites within the transfer configuration.

**Fig. 7:**
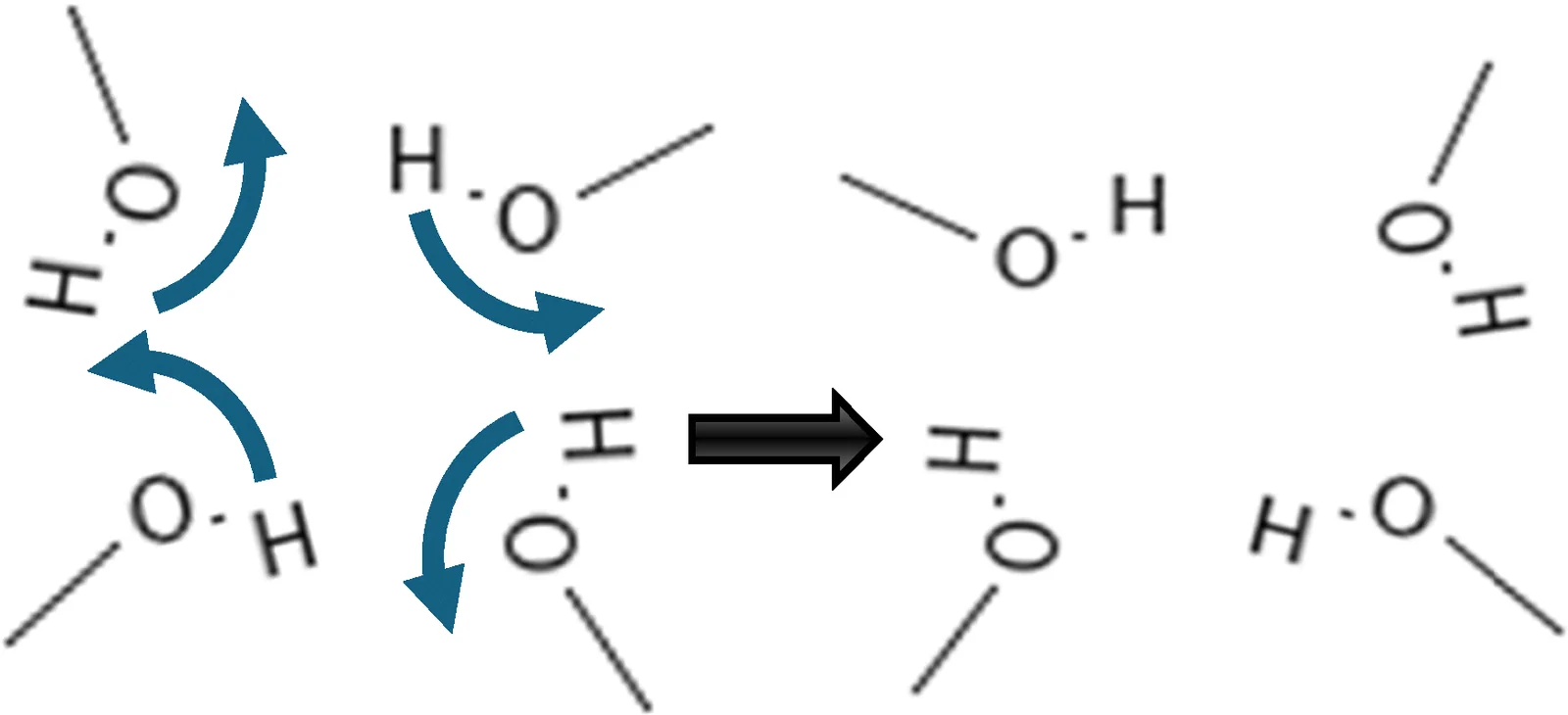
Illustration of the motion of protons within hydroxyl nest defect in zeolites within the rotation configuration.

As discussed in the preceding section, hydroxyl nest defects formed by silicon vacancies in zeolite frameworks exhibit considerable structural flexibility, with hydrogen-bonded silanol groups adopting various configurations. This structural variability implies a complex potential energy surface and suggests that dynamic proton motion plays a central role in the stability and reactivity of these defects. As discussed earlier, two different mechanisms have been proposed to describe proton mobility within hydroxyl nests:

(1) Proton transfer: this mechanism involves the migration of a proton between two neighbouring oxygen atoms within the defect, often referred to as proton hopping, Grotthuss mechanism, proton shuttling, or proton tautomerism in cyclic systems as depicted in Fig. 6. Proton transfer is widely recognised as a key step in the function of Brønsted acid sites and is essential in several catalytic transformations in zeolites, including cracking, isomerization, and dehydration.

(2) Proton rotation: in this alternative mechanism, the proton undergoes rotational motion about a single oxygen atom without full dissociation as depicted in Fig. 7. While energetically less demanding, such rotation can still lead to significant configurational changes within the hydrogen-bond network.

Both processes may proceed *via* classical over-the-barrier dynamics or *via* quantum tunnelling, wherein the proton transitions between equivalent configurations through a potential energy barrier. These phenomena, collectively referred to as proton dynamics, are thought to be integral to the local reactivity and long-range transport processes in zeolitic materials.

### 4.1. NEB simulations of proton motion in chabazite

To explore these proton reorientation mechanisms in detail, we performed nudged elastic band (NEB) simulations within the chabazite (CHA) framework, using the ChemShell software package. As discussed earlier, the NEB method identifies the minimum energy pathway (MEP) between two local minima on the potential energy surface, corresponding to initial and final proton positions. The MEP includes the highest energy point, representing the transition state and activation energy of the process. This approach is well suited to probing rare events such as proton transfer or rotation, where direct molecular dynamics simulations are inefficient.

The initial and final geometries for NEB simulations were generated from previously optimised structures, with the final state defined by relocating a single proton to a new oxygen within the hydrogen-bonded silanol network of the four-membered ring cluster. The fourth silanol group remained unchanged, providing a control for local strain effects. The path between these states was discretized into a set of intermediate images, connected by virtual springs to maintain appropriate spacing. All NEB calculations were performed at the hybrid DFT level with full relaxation of atomic positions perpendicular to the reaction path.

### 4.2. Proton transfer: energetics and geometric response

**Fig. 8:**
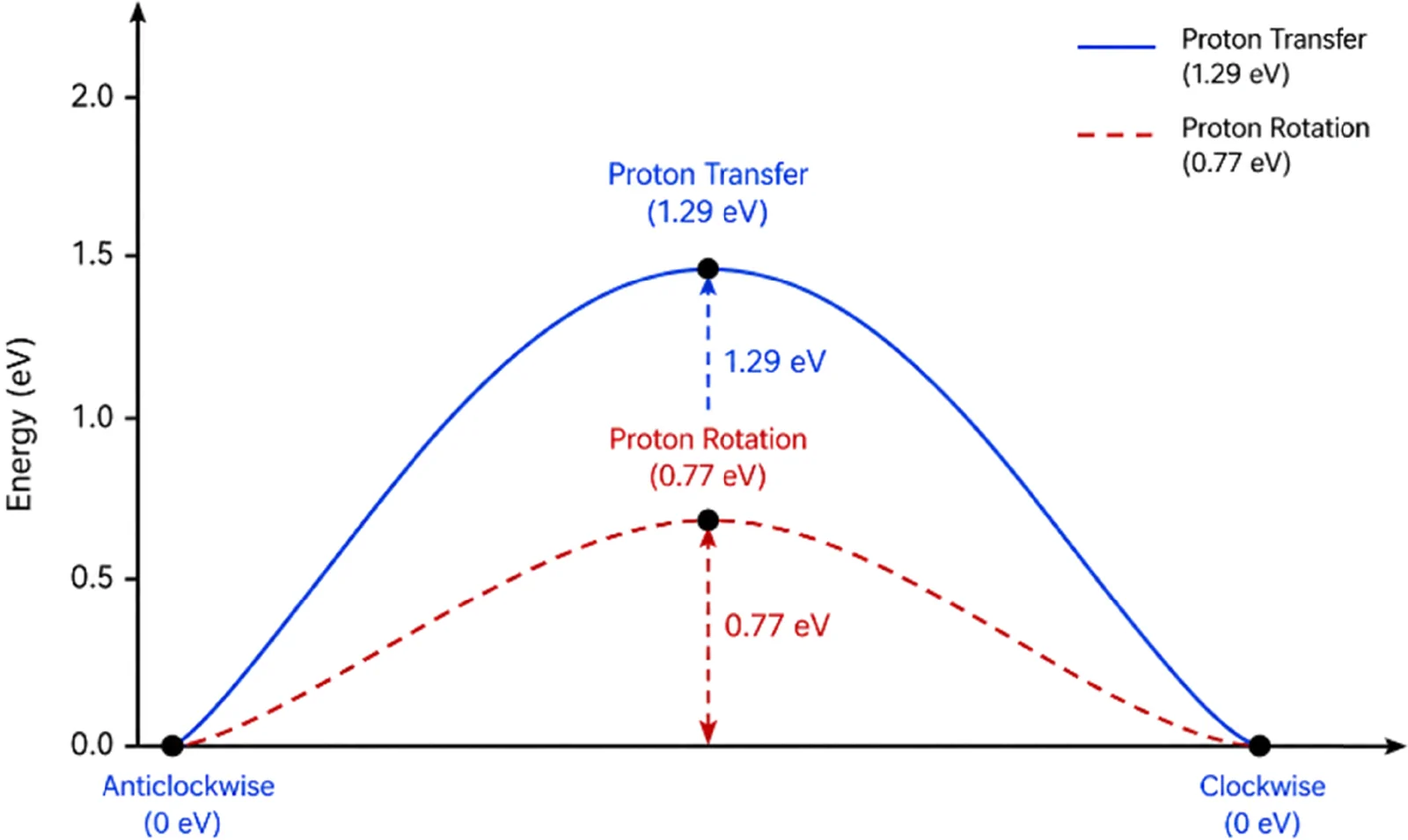
Energy barrier graph showing the energy required to rotate (red) or transfer (blue) a proton between oxygen atoms.

In the proton transfer scenario, the proton migrates between two adjacent silanol oxygen atoms. This process is mechanistically analogous to the Grotthuss mechanism,^34^ whereby proton mobility proceeds not through the physical diffusion of a single hydronium-like species but through a sequential “hopping” of the proton along a chain of hydrogen-bonded oxygen centres, with each transfer step involving the concerted breaking of one O–H bond and formation of a new O–H bond to the neighbouring acceptor. Within the confined hydroxyl nest environment, this hopping is restricted to a single donor acceptor pair rather than propagating along an extended hydrogen bonded chain, but the bond breaking/bond-forming character of the transition state mirrors the elementary step of the classical Grotthuss process. As shown in Fig. 8, the energy profile along the reaction coordinate exhibits a maximum at the fifth image, corresponding to the transition state. Initially, the O–H bond length is 0.970 Å, typical of a well-formed covalent bond. As the proton migrates, the O–H bond to the donor elongates to 1.792 Å, effectively dissociating, before forming a new bond of 0.968 Å with the acceptor oxygen.

The transition state is characterised by a symmetric hydrogen position at an O–H bond length of ∼1.320 Å. The calculated activation energy for this transfer process is 1.29 eV (as depicted in Fig. 8), indicating a substantial barrier imposed by the constraints of the silanol network and associated lattice distortions.

### 4.3. Proton rotation: energetics and geometric response

In contrast, the proton rotation mechanism involves migration of the proton around a single oxygen atom without bond cleavage. Fig. 8 presents the corresponding NEB energy profile, which shows a lower activation barrier of 0.77 eV, approximately 40% lower than for proton transfer. Structural analysis reveals that this rotational motion induces a compression of the O–H bond, reaching a minimum bond length of 0.96 Å at the transition state.

Importantly, all O–H bond lengths throughout the rotation remain within the expected range for hydrogen-bonded silanols, supporting the interpretation that no full dissociation occurs during rotation. The influences of proton hopping and other nuclear quantum effects that occur through proton tunnelling, leading to significantly different reaction rates and mechanisms compared to what classical methods would produce. We note that our results do not capture proton tunnelling effects, which may also have an important influence on the proton rotation. Specifically, protons and other light particles can traverse energy barriers found within the zeolite's structure impacting the kinetics of catalytic reactions, especially in acidic zeolites. This can increase catalytic activity and alter reaction selectivity by providing new reaction pathways that are not available in a purely classical model.^35^

### 4.4. Implications for zeolite function and stability

The NEB simulations presented demonstrate that both proton transfer and proton rotation are feasible dynamic processes within hydroxyl nests in chabazite, though with markedly different energy barriers. The lower barrier for rotation suggests this pathway is energetically preferred under thermal conditions. Furthermore, the rigid CHA framework accommodates these dynamic motions through subtle modulations of local Si–O bond angles and lengths, emphasizing the importance of framework flexibility in enabling proton mobility.

These findings are important for understanding proton dynamics in siliceous frameworks, with implications for proton conduction, catalytic turnover, and the structural evolution of defects under operating conditions. While the current study focuses on the CHA topology, extending this analysis to other zeolitic frameworks such as MFI and FAU will be necessary for generalizing the mechanistic insights.

## 5. Summary and conclusions

This study has presented a comprehensive computational investigation into the formation, structure, and proton dynamics of single hydroxyl nest defects in representative zeolite frameworks: ZSM-5 (MFI), chabazite (CHA), faujasite (FAU), and α-quartz. Using hybrid QM/MM embedding and density functional theory, we have investigated both the thermodynamic stability, and the geometric signatures associated with these defects, as well as possible framework-dependent mechanisms for proton motion within them.

Analysis of proton dynamics within the chabazite framework reveals two distinct and competing mechanisms: proton transfer between silanol groups and proton rotation about a single oxygen. The significantly lower activation barrier for rotational motion (0.77 eV) compared to transfer (1.29 eV) indicates that local reorientation dominates under typical conditions, while long-range proton relocation *via* hopping is kinetically limited. This distinction is critical, as it suggests that hydroxyl nests primarily enable rapid local restructuring of hydrogen-bond networks rather than efficient long-range proton transport.

These findings have direct implications for zeolite functionality. Dynamic proton rearrangement within hydroxyl nests may modulate the strength, accessibility, and cooperativity of Brønsted acid sites, particularly under conditions where hydrogen-bonded networks are stabilised. As such, hydroxyl nests should be viewed not only as structural defects but as dynamic entities that can influence catalytic turnover, adsorption behaviour, and proton-mediated processes as observed in literature previously stated. The strong dependence of both defect energetics and proton mobility on framework geometry further emphasises that these effects are highly topology specific.

## Author contributions

All authors contributed to work presented in this paper. The initial draft of the paper was written by AWD and was subsequently modified by the other authors.

## Conflicts of interest

There are no conflicts to declare.

## Data Availability

All data for zeolite structures and results are available on NOMAD-LAB website: FAU – https://nomad-lab.eu/prod/v1/gui/user/uploads/upload/id/lWOhsNZgTF-KZSjUtiv2mA; Alpha quartz – https://nomad-lab.eu/prod/v1/gui/user/uploads/upload/id/fLtSoEMBQiie15RRsdk2ow; CHA – https://nomad-lab.eu/prod/v1/gui/user/uploads/upload/id/0xCSOtd2Qmm2_yT8BL8UZQ; ZSM-5 – https://nomad-lab.eu/prod/v1/gui/user/uploads/upload/id/tT0Hk8NXT1yLVeaycgg8ZQ.

## References

[cit1] Salahudeen N. (2022). A Review on Zeolite: Application, Synthesis and Effect of Synthesis Parameters on Product Properties. Chem. Afr..

[cit2] Marcilly C. R. (2000). Where and how shape selectivity of molecular sieves operates in refining and petrochemistry catalytic processes. Top. Catal..

[cit3] Corma A. (2000). *et al.*, Preparation, characterisation and catalytic activity of ITQ-2, a delaminated zeolite. Microporous Mesoporous Mater..

[cit4] Primo A., Garcia H. (2014). Zeolites as catalysts in oil refining. Chem. Soc. Rev..

[cit5] Lee E. F. T., Rees L. V. C. (1987). Dealumination of sodium Y zeolite with hydrochloric acid. J. Chem. Soc., Faraday Trans. 1.

[cit6] Wang Y. (2016). *et al.*, Effects of Dealumination and Desilication of Beta Zeolite on Catalytic Performance in n-Hexane Cracking. Catalysts.

[cit7] Weitkamp J. (2000). Zeolites and catalysis. Solid State Ionics.

[cit8] Kresge C. T., Leonowicz M. E., Roth W. J., Vartuli J. C., Beck J. S. (1992). Ordered mesoporous molecular sieves synthesized by a liquid-crystal template mechanism. Nature.

[cit9] Donaldson P. M., Hawkins A. P., Howe R. F. (2025). Distinctive signatures and ultrafast dynamics of Brønsted sites, silanol nests and adsorbed water in zeolites revealed by 2D-IR spectroscopy. Chem. Sci..

[cit10] Ray G. J., Nerheim A. G., Donohue J. A. (1988). Characterization of defects in dealuminated faujasite. Zeolites.

[cit11] Ray G. J. (1992). *et al.*, Silanol chemistry in siliceous faujasite. Colloids Surf..

[cit12] Barrer R. M., Makki M. B. (1964). Molecular sieve sorbents from clinoptilolite. Canadian J. Chem..

[cit13] Sokol A. A., Catlow C. R. A., Garcés J. M., Kuperman A. (2004). Transformation of hydroxyl nests in microporous aluminosilicates upon annealing. J. Phys.: Condens. Matter.

[cit14] Sokol A. A., Catlow C. R. A., Garcés J. M., Kuperman A. (2002). Local States in Microporous Silica and Aluminum Silicate Materials. 1. Modeling Structure, Formation, and Transformation of Common Hydrogen Containing Defects. J. Phys. Chem. B.

[cit15] Colella C., Gualtieri A. F. (2007). Cronstedt's zeolite. Microporous Mesoporous Mater..

[cit16] Schroeder C. (2020). *et al.*, Disentangling Brønsted Acid Sites and Hydrogen-Bonded Silanol Groups in High-Silica Zeolite H-ZSM-5. J. Phys. Chem. C.

[cit17] Schröder K.-P. (1992). *et al.*, Siting of AI and bridging hydroxyl groups in ZSM-5: A computer simulation study. Zeolites.

[cit18] Sauer J., Zahradník R. (1984). Quantum chemical studies on zeolites and silica. Int. J. Quantum Chem..

[cit19] Catlow C. R. A. , et al., Modelling of structure and reactivity in zeolites, in Studies in Surface Science and Catalysis, ed. L. Bonneviot and S. Kaliaguine, 1995, Elsevier, pp. 87–100

[cit20] Metz S. (2014). *et al.*, ChemShell-a modular software package for QM/MM simulations. Wiley Interdiscip. Rev.: Comput. Mol. Sci..

[cit21] Lu Y., Farrow M. R., Fayon P., Logsdail A. J., Sokol A. A., Catlow C. R. A., Sherwood P., Keal T. W. (2019). Open-Source, Python-Based Redevelopment of the ChemShell Multiscale QM/MM Environment. J. Chem. Theory Comput..

[cit22] Valiev M. (2010). *et al.*, NWChem: A comprehensive and scalable open-source solution for large scale molecular simulations. Comput. Phys. Commun..

[cit23] Smith W., Yong C. W., Rodger P. M. (2002). DL_POLY: Application to molecular simulation. Mol. Simul..

[cit24] Gale J. D., Rohl A. L. (2003). The General Utility Lattice Program (GULP). Mol. Simul..

[cit25] Shu-Meng H. (2019). *et al.*, Hierarchical mesoporous cobalt silicate architectures as high-performance sulfate-radical-based advanced oxidization catalysts. J. Colloid Interface Sci..

[cit26] Boulfelfel S. E. (2016). *et al.*, Improved Hill–Sauer Force Field for Accurate Description of Pores in 8-Ring Zeolites. J. Phys. Chem. C.

[cit27] P. Sherwood, Vries A. H. D., Collins S. J., Greatbanks S. P., Burton N. A., Vincent M. A., Hillier I. H. (1997). Computer simulation of zeolite structure and reactivity using embedded cluster methods. Faraday Discuss..

[cit28] Becke A. D. (1993). A new mixing of Hartree–Fock and local density-functional theories. J. Chem. Phys..

[cit29] Weigend F., Ahlrichs R. (2005). Balanced basis sets of split valence, triple zeta valence and quadruple zeta valence quality for H to Rn: Design and assessment of accuracy. Phys. Chem. Chem. Phys..

[cit30] Henkelman G., Uberuaga B. P., Jónsson H. (2000). A climbing image nudged elastic band method for finding saddle points and minimum energy paths. J. Chem. Phys..

[cit31] Kästner J., Carr J. M., Keal T. W., Thiel W., wander A., Sherwood P. (2009). DL-FIND: An Open-Source Geometry Optimizer for Atomistic Simulations. J. Phys. Chem. A.

[cit32] Wright K., Freer R., Catlow C. R. A. (1994). The energetics and structure of the hydrogarnet defect in grossular: A computer simulation study. Phys. Chem. Miner..

[cit33] Lin J. S. (1994). *et al.*, Ab initio calculations on (OH)4 defects in α-quartz. Phys. Chem. Miner..

[cit34] Noam A. (1995). The Grotthuss mechanism. Chem. Phys. Lett..

[cit35] Truong T. N. (1997). Thermal Rates of Hydrogen Exchange of Methane with Zeolite:  A Direct ab Initio Dynamics Study on the Importance of Quantum Tunneling Effects. J. Phys. Chem. B.

